# Interleukin-32α promotes the proliferation of multiple myeloma cells by inducing production of IL-6 in bone marrow stromal cells

**DOI:** 10.18632/oncotarget.21611

**Published:** 2017-10-07

**Authors:** Xuanru Lin, Li Yang, Gang Wang, Fuming Zi, Haimeng Yan, Xing Guo, Jing Chen, Qingxiao Chen, Xi Huang, Yi Li, Enfan Zhang, Wenjun Wu, Yang Yang, Donghua He, Jingsong He, Zhen Cai

**Affiliations:** ^1^ Bone Marrow Transplantation Center, The First Affiliated Hospital, School of Medicine, Zhejiang University, Hangzhou, Zhejiang, China; ^2^ Department of Hematology, People's Hospital of Quzhou, Quzhou, Zhejiang, China; ^3^ Department of Hematology, The Second Affiliated Hospital of Nanchang University, Nanchang, Jiangxi, China

**Keywords:** interleukin-32, IL-6, multiple myeloma, bone marrow stromal cells

## Abstract

Multiple myeloma (MM) is a malignant plasma disease closely associated with inflammation. In MM bone marrow microenvironment, bone marrow stromal cells (BMSCs) are the primary source of interleukin-6 (IL-6) secretion, which promotes the proliferation and progression of MM cells. However, it is still unknown how the microenvironment stimulates BMSCs to secrete IL-6. Interleukin-32 (IL-32) is a newly identified pro-inflammatory factor. It was reported that in solid tumors, IL-32 induces changes in other inflammatory factors including IL-6, IL-10, and TNF-α. The aim of this study was to investigate the expression of IL-32 and the role of IL-32 in the MM bone marrow microenvironment. Our data illustrate that MM patients have higher expression of IL-32 than healthy individuals in both bone marrow and peripheral blood. We used ELISA and qRT-PCR to find that malignant plasma cells are the primary source of IL-32 production in MM bone marrow. ELISA and Western blot analysis revealed that recombinant IL-32α induces production of IL-6 in BMSCs by activating NF-κB and STAT3 signaling pathways, konckdown of IL-32 receptor PR3 inhibit this process. Knockdown of IL-32 by shRNA decreased the proliferation in MM cells that induced by BMSCs. In conclusion, IL-32 secreted from MM cells has paracrine effect to induce production of IL-6 in BMSCs, thus feedback to promote MM cells growth.

## INTRODUCTION

Multiple myeloma (MM), characterized by the accumulation of malignant plasma cells and the production of monoclonal immunoglobulin, is an incurable malignant blood disease that originates from the B cell lineage and accounts for approximately 21% of deaths from hematological malignancies in the US [1–3]. MM development is primarily dependent on the bone marrow (BM) microenvironment. Direct contact with cells in the BM microenvironment, such as bone marrow stromal cells (BMSCs), macrophages, osteoclasts and endothelial cells, induces MM cell growth, proliferation, invasion and metastasis, as do the cytokines and the chemokines in the microenvironment [4–6]. MM is closely associated with inflammation. Patients with autoimmune disease, a history of infection, and other inflammatory diseases have a higher incidence of MM and monoclonal gammopathy of undetermined significance (MGUS) [7]. Researches showed that inflammatory mediators, such as interleukin 6 (IL-6), Toll-like receptor (TLR), signal transducer and activator of transcription 3 (STAT3), and nuclear factor-κB (NF-κB), are critical in the transformation from inflammation to tumor [8, 9]. Previous studies have demonstrated that MM cells are modified by BMSCs that form an inflammatory microenvironment that benefits the MM cells in the BM [10, 11]. Jego et al. reported that IL-6, which is mainly produced by BMSCs, plays a key role in the proliferation and progression induced by repeated infections in MM [12]. However, how inflammation induces BMSCs in the MM BM microenvironment to secrete IL-6 remains unknown.

Recent studies have indicated that interleukin 32 (IL-32), a newly defined pro-inflammatory cytokine that was initially discovered from natural killer cells and is also named natural killer 4 (NK-4) [13], increases the secretion of inflammatory factors such as Interleukin-6 (IL-6), IL-1β and TNF-α, thus inducing an inflammation ‘cascade amplification effect’ [14, 15].

IL-32 is a unique cytokine with different isoforms that are required to activate specific cells and have diverse effects on different cells [16–18]. Studies in solid tumors, such as gastric cancers, pancreatic cancers and renal carcinomas, have found high expression of IL-32, suggesting that this cytokine is an independent prognostic factor [19–21]. Howener, in HeLa cells, overexpression of IL-32 increases cell apoptosis, and impairs cell growth and migration [22]. Moreover, in hematological malignancies, IL-32 is remarkably increased in patients with myelodysplastic syndrome (MDS), whereas patients with chronic myelomonocytic leukemia (CMML) have markedly reduced expression of IL-32 [23]. Thus, the role and function of IL-32 in tumors are still controversial. In addition, the expression of IL-32 in MM remains uncovered, and it is still unknown whether IL-32 has a pro-inflammatory function in the MM BM microenvironment formed by BMSCs and other cells.

In this study, we analyzed the expression of IL-32 in MM cells and in the MM BM microenvironment, and explored the role and mechanism of IL-32 in the inflammatory factors network and the proliferation of MM cells.

## RESULTS

### Expression of IL-32 in human myeloma cells and BMSCs

**Figure 1:**
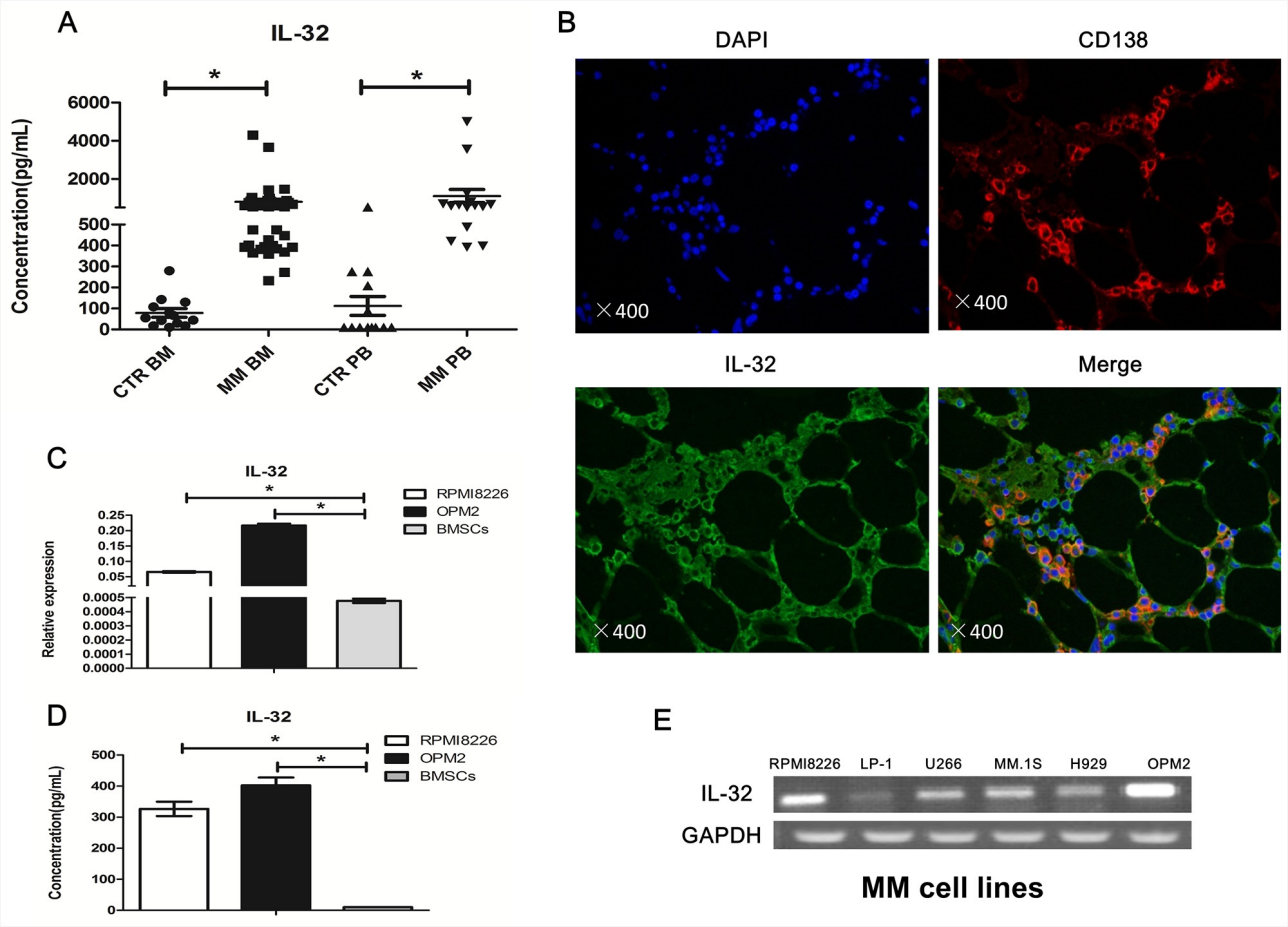
Expression of IL-32 in MM patients, human myeloma cells and BMSCs **(A)** Expression of IL-32 in BM and PB, MM patients are compared to normal healthy donors (CTR), measured by ELISA. **(B)** Immunofluorescence analysis of MM patients BM biopsies with anti-CD138, anti-IL-32 antibodies (magnification: ×400). **(C)** PCR analysis of the expression of IL-32 in RPMI8226, OPM2 and BMSCs. BMSCs obtained from different MM patient (n=9). **(D)** Expression of soluble IL-32 in RPMI8226, OPM2 and BMSCs, measured by ELISA; BMSCs obtained from different MM patient (n=9). **(E)** PCR analysis of the expression of IL-32 in MM cell lines. ^*^ *p*<0.05.

As a cytokine, we first detected soluble IL-32 in the BM of both newly diagnosed MM patients (n=38) and normal healthy donors (n=13) by ELISA. As shown in Figure 1A, MM patients exhibited significantly higher secretion of soluble IL-32 compared to healthy controls (798.2±132.1pg/mL vs 79.88±20.54pg/mL, *p*<0.05). In peripheral blood (PB), the expression of IL-32 in MM patients (n=15) was higher than that in healthy donors (n=15) as well (1103±344.7pg/mL vs 111.8±45.13pg/mL, *p*<0.05) (Figure 1A). Next, immunofluorescence analysis of human BM biopsies showed that both CD138^+^ and CD138^−^ cells in MM BM express IL-32 (Figure 1B). Finally, we checked the expression of IL-32 in MM cell lines and human BMSCs using qRT-PCR and ELISA. The results suggested that MM cell lines also expressed IL-32. Some of the cell lines like LP-1, H929 expressed relatively low IL-32, while RPMI8226 and OPM2 had extremely high expression of IL-32 among the rest (Figure 1E). RPMI8226 and OPM2 express approximately 50-100 folds higher levels of IL-32 mRNA than BMSCs (Figure 1C, 1D).

**Figure 2:**
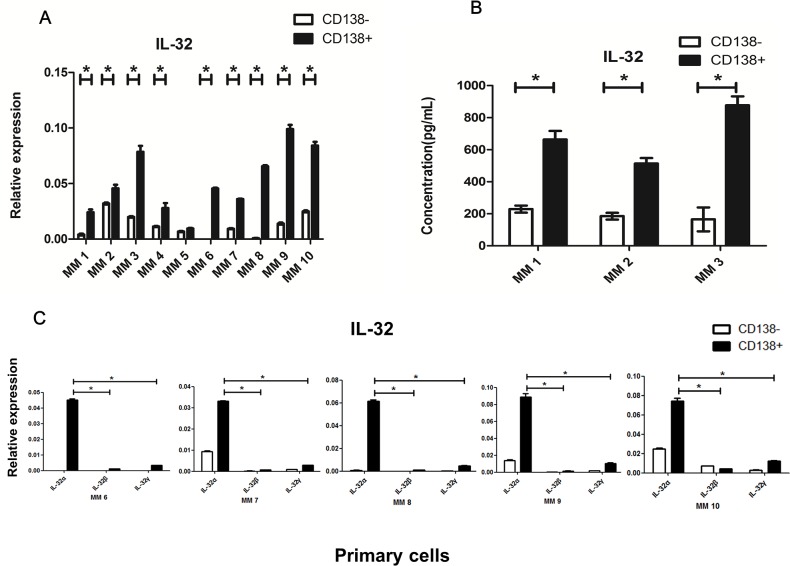
Expression of IL-32 in primary MM cells **(A)** qRT-PCR analysis of the expression of IL-32 in primary CD138^+^ and CD138^−^ cells isolated from MM patients. **(B)** Primary CD138^+^ and CD138^−^ cells isolated from MM patients were cultured *in vitro* for 12h (inclusion criteria: cell apoptosis<50%, detected by flow cytometry), ELISA was applied to detect soluble IL-32 in the culture medium. **(C)** qRT-PCR analysis of the expression of different IL-32 isoforms (IL-32α, IL-32β, IL-32γ) in primary CD138^+^ and CD138^−^ cells isolated from MM patients. ^*^ *p*<0.05.

As the MM BM microenvironment contains both CD138^+^ malignant plasma cells and CD138^−^ non-plasma cells, we tried to examine the cell sources that contributed to the majority of the IL-32 secretion. qRT-PCR was used to assess gene expression, and ELISA was applied for the detection of soluble IL-32 in primary CD138^+^ cells and CD138^−^ cells isolated from 10 newly diagnosed untreated MM patients. The former, considered as primary MM cells, had a relatively high expression of IL-32 compared to the latter (Figure 2A, 2B). As IL-32 has different isoforms, furthermore, we detected 5 samples from the above by qRT-PCR, found that the most prevalent expressed isoform in primary MM cells was IL-32α (Figure 2C).

### IL-32α induces production of IL-6 in MM BMSCs

**Figure 3:**
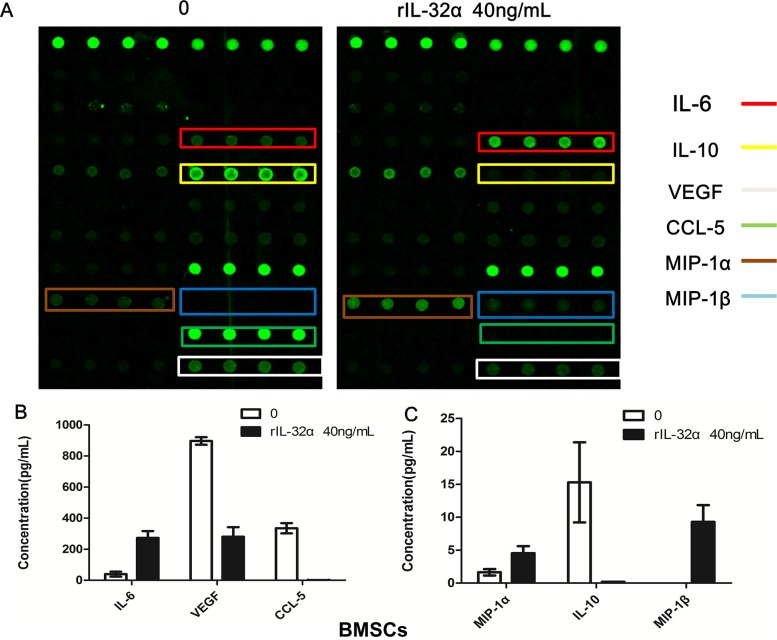
Cytokine array analysis in BMSCs induced by rIL-32α **(A)** Cytokines and chemokines changes in BMSCs with or without 40 ng/mL rIL-32α stimulation. These images show the results of BMSCs obtained from one patient. **(B) (C)** Total analysis of cytokines and chemokines in BMSCs obtained from different patients (n=4).

To determine the effect of IL-32 in the MM BM microenvironment, a cytokine array was applied to assay cytokines and chemokines induced or reduced by rIL-32α. As shown in Figure 3, not only the production of IL-6 in BMSCs, but also the expression of MIP-1α and MIP-1β increased. In addition, CCL-5, IL-10 and VEGF decreased.

**Figure 4:**
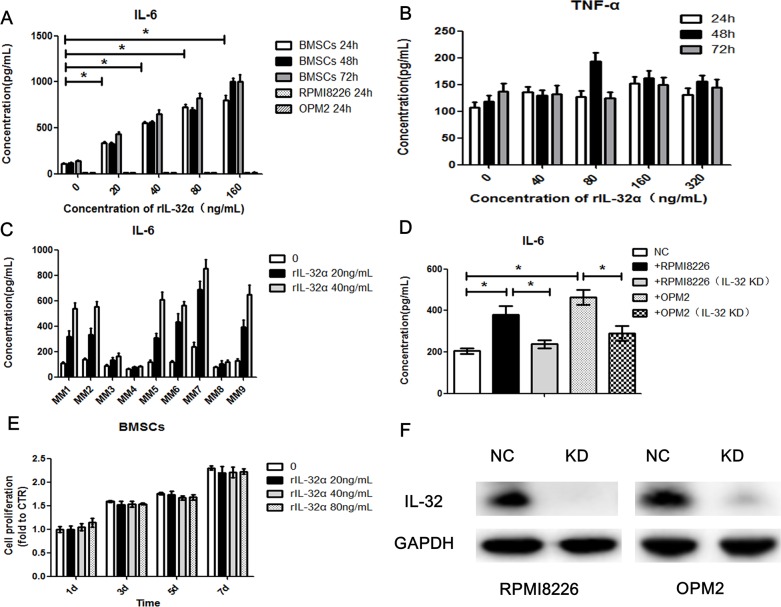
IL-32α induces production of IL-6 in MM BMSCs **(A)** Concentration of IL-6 in BMSCs in the presence of 0-160 ng/mL rIL-32α, stimulated for 24, 48, and 72 h. BMSCs obtained from one MM patient, repeated in three independent experiments, measured by ELISA. **(B)** Concentration of TNF-α in BMSCs in the presence of 0-320 ng/mL rIL-32α, stimulated for 24, 48 and 72 h. Samples obtained from one MM patient, repeated in three independent experiments, measured by ELISA. **(C)** Concentration of IL-6 in BMSCs in the presence of 0-40 ng/mL rIL-32α, stimulated for 24 h, BMSCs obtained from different patients, and 6 in 9 (66.7%) showed positive effects, measured by ELISA. **(D)** Concentration of IL-6 in BMSCs co-cultured with IL-32 high-expression MM cell lines, with or without IL-32 knockdown; BMSCs were co-cultured with MM cells in a 24-well Transwell plate, repeated in three independent experiments, measured by ELISA. **(E)** Cell proliferation of BMSCs in the presence of 0-80 ng/mL rIL-32α, stimulated for 1, 3, 5 and 7 d; cells were cultured in 96 well plates, repeated in three independent experiments, measured by CCK8 assay. **(F)** Identification of IL-32 knockdown MM cell lines, Western blot analysis.

To verify the result of the cytokine array, we stimulated BMSCs with rIL-32α in a concentrarion escalating manner (20ng/mL, 40ng/mL, 80ng/mL and 160ng/mL) and in a time escalating manner (24 h, 48 h and 72 h). ELISA was applied to detect soluble IL-6 secreted into the culture media. As shown in Figure 4A, compared to control, 20ng/mL rIL-32α significantly induced BMSCs to express IL-6 (e.g., at 24 h, 335.3±11.2pg/mL vs 106.8±9.7pg/mL, *p*<0.05). As the concentration of rIL-32α increased, the release of IL-6 by BMSCs achieved higher levels approximately 3 to 8 fold higher than normal. However, there were no specific time dependent changes (*p*>0.05) (Figure 2A), and rIL-32α did not induce TNF-α secretion in BMSCs (Figure 4B). As BMSCs have a basement secretion of IL-6, we tried to determine whether the increase in IL-6 secretion was due to the proliferation of BMSCs. We used CCK8 assay to evaluate the growth of BMSCs. The results showed that rIL-32α did not promote the proliferation of BMSCs directly (Figure 4E). Further, we examined BMSCs obtained from different individuals (n=9), and 66.7% showed a positive effect in the stimulation of rIL-32α with an appropriate concentration (20ng/mL, 40ng/mL) for 24 h (Figure 4C). We also stimulated MM cell lines with rIL-32α. MM cell lines were not expected to secrete as much IL-6 as BMSCs, nor did they when stimulated with rIL-32α (Figure 2A).

As IL-32 may be primarily from the plasma cells, we next co-cultured BMSCs with MM cell lines (BMSCs in the lower chamber, MM cell lines in the Transwell inserts, pore size: 0.4μm) to determine the functions of MM cells secreted IL-32. We compared normal MM cells to IL-32-knockdown MM cells and found that RPMI8226 cells induced BMSCs to express and secrete higher levels of IL-6 (360.8±28.4pg/mL vs 177.3±9.4pg/mL, *p*<0.05). Knockdown of IL-32 in MM cells by shRNA reduced the enhancing effect toward BMSCs (246.7±10.9 pg/mL vs 360.8±28.4 pg/mL, *p*<0.05). Similar results were observed in OPM2 cells as well as in RPMI8226 cells (Figure 4D).

### IL-32α activates the NF-κB and STAT3 signaling pathways in BMSCs

Studies have demonstrated that, in the MM BM microenvironment, NF-κB and STAT3 transcriptional activities are induced by cytokines, and then collaborate to induce IL-6.

**Figure 5:**
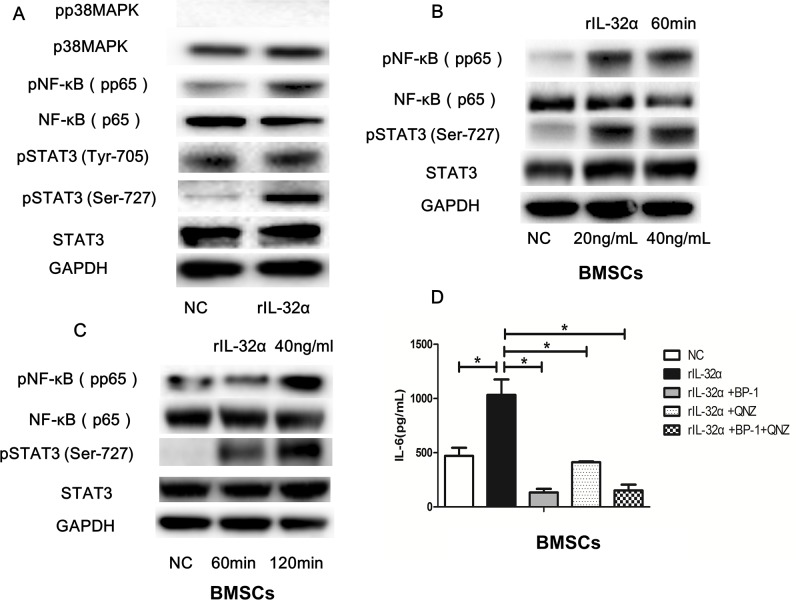
IL-32α activates the NF-κB and STAT3 signaling pathways in BMSCs **(A)** Western blot analysisof p38, NF-κB and STAT3 signaling pathway and in BMSCs with or without 40 ng/mL rIL-32α stimulation for 60 min. **(B)** Western blot analysisof NF-κB and STAT3 signaling pathwaysin BMSCs with 20-40 ng/mL rIL-32α stimulation for 60 min. **(C)** Western blot analysisof NF-κB and STAT3 signaling pathwaysin BMSCs with 40 ng/mL rIL-32α stimulation for 60-120 min. **(D)** Concentration of IL-6 in BMSCs in the presence of 40 ng/mL rIL-32α for 24 h, with or without NF-κB inhibitor QNZ (10μM/mL), STAT3 inhibitor BP-1-102 (10μM/mL), measured by ELISA; BMSCs obtained from one MM patient, repeated in three independent experiments. ^*^ *p*<0.05.

Western blotting was applied to detect a possible inflammation signaling pathway in BMSCs induced by rIL-32α. As shown in Figure 5A, NF-κB and STAT3 pathways were activated through upregulation of the phosphorylation of p65NF-κB and STAT3 Ser-727, but no significant changes in the phosphorylation of STAT3 Tyr-705 or p38MAPK were observed. Subsequently, we detected the phosphorylation of p65NF-κB and STAT3 Ser-727 induced by rIL-32α in a concentration-escalating manner (20 ng/mL and 40 ng/mL) and in a time-escalating manner (1 h and 2 h). The results showed that phosphorylation of p65NF-κB and STAT3 Ser-727 increased as the concentration of rIL-32α rose, and the stimulation was consistent (Figure 5B, 5C). To confirm that, we used NF-κB inhibitor QNZ (10μM/mL) and STAT3 inhibitor BP-1-102 (10μM/mL) to block the activation of these two inflammation signaling pathways. As shown in Figure 5D, rIL-32α+QNZ treated group and rIL-32α+BP-1-102 treated group both decreased the production of IL-6 in BMSCs compared to the group that treated with rIL-32α alone (411.75±5.39 pg/mL, 133.18±23.65 pg/mL vs 1033.12±100.71 pg/mL, *p*<0.05).

### Expression of PR3 in BMSCs

**Figure 6:**
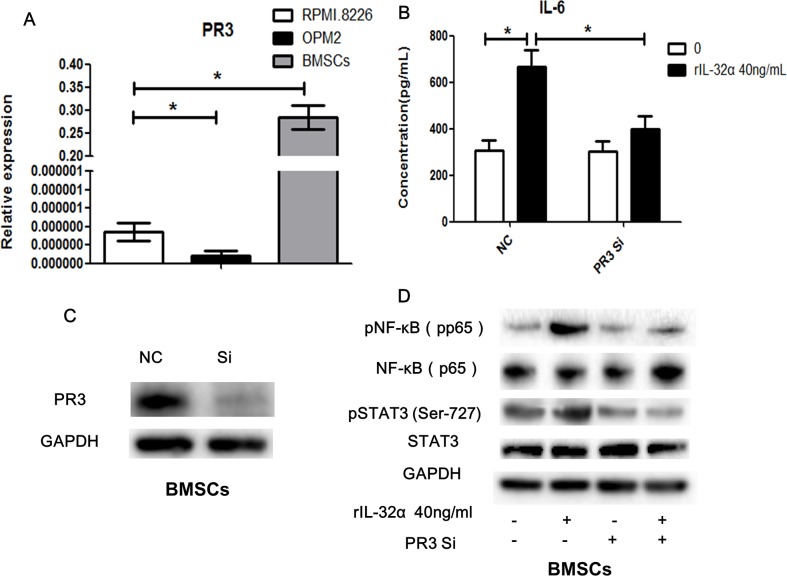
PR3 involved in IL-32 induced IL-6 production in BMSCs **(A)** qRT-PCR analysis of the expression of PR3 in BMSCs and MM cells. **(B)** Concentration of IL-6 in BMSCs in the presence of 40 ng/mL rIL-32α for 24 h, with or without PR3 knockdown, BMSCs obtained from one MM patient, repeated in three independent experiments, measured by ELISA. **(C)** Identification of PR3 knockdown BMSCs. **(D)** Western blot analysisof NF-κB and STAT3 signaling pathwaysin BMSCs in the presence of 20 ng/mL rIL-32α for 60min, with or without PR3 knockdown. ^*^ *p*<0.05.

We examined the expression of proteinase 3 (PR3), a binding protein of extracellular IL-32 that increase its activity, using qRT-PCR. As shown in Figure 6A, BMSCs express significant higher levels of PR3 mRNA compared to MM cell lines.

To verify the participation of the NF-κB and STAT3 signaling pathways in IL-32 and BMSCs-mediated production of IL-6, we inhibited the expression of PR3 in BMSCs using siRNA. In the BMSCs group with PR3 knockdown, the production of IL-6 after stimulation with rIL-32α decreased markedly compared to the group without PR3 knockdown (398.6±56.92 pg/mL vs 665.7±73.13 pg/mL, 24 h, *p*<0.05), but it was still higher than the group that was not stimulated (306.6±42.14 pg/mL) (Figure 6B). Similarly, knockdown of PR3 reduced the activation of the NF-κB and STAT3 signaling pathways induced by rIL-32α compared to the group without PR3 knockdown (Figure 6D).

### IL-32α promoted the proliferation of MM cells in the tumor microenvironment

**Figure 7:**
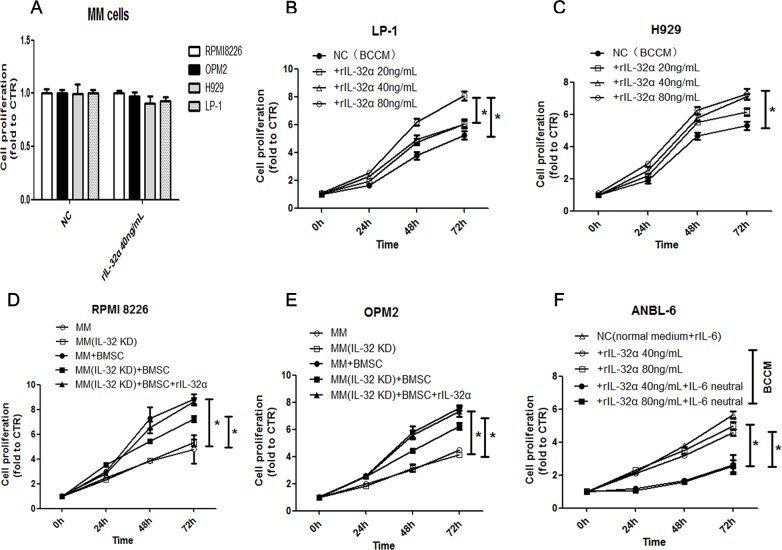
IL-32α promotes the proliferation of MM cells in the tumor microenvironment **(A)** Cell proliferation of MM cell lines during exposure to 40 ng/mL rIL-32α for 24 h. Repeated in three independent experiments, measured by CCK8. **(B) (C)** Cell proliferation in IL-32 low-expression MM cell lines, H929 and LP-1, cultured in BCCM stimulated by 0-80 ng/mL rIL-32α for 24, 48 and 72 h. MM cells were cultured in 96-well plates. Repeated in three independent experiments, measured by CCK8. **(D) (E)** Cell proliferation in IL-32 high-expression MM cell lines, RPMI8226 and OPM2, cultured alone or co-cultured with BMSCs for 24, 48, and 72 h, with or without IL-32 knockdown. rIL-32α rescue concentration was 40ng/mL. MM cells were co-cultured with BMSCs in 24-well plates and transferred to 96-well plates to be assayed. Repeated in three independent experiments, measured by CCK8. **(F)** Cell proliferation in IL-6 dependent MM cell line ANBL-6, cultured in BCCM stimulated by 0-80 ng/mL rIL-32α for 24, 48 and 72 h, with or without IL-6 neutralization antibodies (20ng/mL); cells in control group were cultured in normal medium with rIL-6 (20ng/mL); MM cells were cultured in 96-well plates. Repeated in three independent experiments, measured by CCK8.

The role of IL-6 in MM as a growth and survival factor is unambiguous. As IL-32 induced the production of IL-6 in BMSCs, we tried to verify the biological function of IL-32 toward MM cells in the BM microenvironment. First, rIL-32α was used to stimulate both IL-32 low-expression and high-expression MM cell lines when cultured with normal medium, it seemed not to promote the proliferation of MM cells directly (Figure 7A), nor did we detect any changes in apoptosis (data not shown). Second, BMSCs conditioned medium (BCCM) was used to culture IL-32 low-expression MM cell lines (H929 and LP-1) for 72 h and CCK8 assay was applied to analyze the proliferation of MM cells. The rIL-32α-treated BCCM significantly promoted the proliferation of H929 cells and LP-1 cells compared to the non-treated BCCM (e.g., H929, rIL-32α 40ng/mL 72h, 7.11±0.18 vs 5.32±0.26, *p*<0.05) (Figure 7B, 7C, Supplementary Figure 1). Then, we co-cultured IL-32 high-expression MM cell lines (RPMI8226 and OPM2) with BMSCs as mentioned above with or without knockdown of IL-32. As shown in Figure 7D and 7E, IL-32-knockdown RPMI8226 cells and OPM2 cells showed a lower proliferation rate in the imitated BM microenvironment compared to normal MM cells (e.g., RPMI8226, 72 h,7.24±0.20 vs 8.82±0.34, *p*<0.05); when rIL-32α (40ng/mL) was applied to rescue the IL-32-knockdown MM cells in the co-culture system, the proliferation rate increased again (e.g., RPMI8226, 72 h,8.62±0.26 vs 7.24±0.20, *p*<0.05). However, there was not much difference in growth between the IL-32-knockdown cell lines and the normal cell lines when cultured alone. Finally, to confirm that the increased proliferation was due to the production of IL-6 in BMSCs, we repeated the experiments that were applied for LP-1 and H929 before in IL-6 dependent MM cell line ANBL-6, the result (Figure 7F) showed that rIL-32α-treated BCCM had nearly the same effect compared to normal medium with rIL-6 (20ng/mL) (e.g., ANBL-6, rIL-32α 40ng/mL 72h, 4.60±0.17 vs 5.65±0.18), IL-6 neutralization antibodies (20ng/mL) significantly inhibited the proliferation of ANBL-6 cells cultured in BCCM (e.g., ANBL-6, rIL-32α 40ng/mL+IL-6 neutral 72h, 2.64±0.51 vs 4.60±0.17, *p*<0.05). These data suggested that IL-32 may be more likely to have paracrine effects in the BM microenvironment than MM cells themselves, and then a feedback mechanism supports MM cells.

## DISCUSSION

IL-32 is a novel pro-inflammatory cytokine that is found to exist in nine different isoforms, including IL-32α, IL-32β, IL-32γ, IL-32δ, IL-32ε, IL-32ζ, IL-32η and IL-32θ [16]. Kim et al. first reported its biological function in 2005 [24]. Accumulatiing evidence indicates that IL-32 is a pluripotent cytokine with diverse functions [25–28]. NK-4 coding protein was initially assumed to be a secreted protein, but some newly found isoforms lack of a putative signal peptide, and the amount secreted from cells is small [16, 17]. Hasegawa reported that IL-32 might be released from intestinal epithelial cells via a non-classical secretory route, such as multi-vesicular and exosomal secretion [29]. Studies have also demonstrated that intracellular IL-32 interferes with Paxillin-FAK binding by resembling the FAT region, where as extracellular IL-32 binds to PR3 [30]. Therefore, it seems that IL-32 has both intracellular and extracellular biological functions according to the differential uptake by cells and the different isoforms.

Numerous studies have been published on the role of IL-32 in cancers and cancer- related malignancies. It is still controversial because IL-32 exhibits opposite effects in different tumors. In human stomach cancer and pancreatic cancer patients, IL-32 is highly expressed compared with healthy individuals [20, 31]. In renal cell carcinoma, a previous study showed that IL-32 overexpression was associated with high recurrence rates, low recurrence free survival and overall survival, and thus may be a new prognostic factor [21]. Tsai and co-workers also reported that high expression of IL-32 in gastric cancer was positively correlated with tumor metastasis and poor prognosis [19]. In this study, we examined the expression of IL-32 in peripheral blood and bone marrow and found that MM patients have higher expression of IL-32 compared to healthy individuals. Further, we confirmed that the production of IL-32 in MM patients was primarily from the CD138^+^ malignant plasma cells rather than the CD138^−^ cells in the BM microenvironment. In addition, IL-32 is also associated with inflammatory diseases and autoimmune diseases. In COPD, it was reported that IL-32 was linked to pathologic changes in patients. Another study demonstrated that in serum from myasthenia gravis patients, IL-32α was significantly increased [32]. As MM is closely related with autoimmune disease and chronic inflammation, high expression of IL-32 in MM cells may also contribute to the evolution of malignant plasma cells, which needs to be thoroughly explored.

IL-32α is the most abundant clone among all the IL-32 isoforms, and it is expressed in various cells such as NK cells, T cells and monocytes [24], we also found that the majority expressed in the primary MM cells was IL-32α. Studies have demonstrated that, unlike IL-32β and IL-32γ, over-expression of IL-32α does not cause cell death directly but increases the NK cell-mediated killing in chronic myeloid leukemia cells [33, 34]. More importantly, intracellular IL-32α induces STAT3 Ser-727 phosphorylation by binding with PMA-activated PKCε and STAT3 and thus enhances IL-6 production [35], while the other two isoforms, IL-32β and IL-32γ, inactivate the STAT3 signaling pathway [36, 37]. On the other hand, PR3 binds specifically to extracellular IL-32α with high affinity [30, 38]. For the purpose of identifying the function of IL-32α as a secreted cytokine in MM, our studies showed that rIL-32α induced the production of IL-6 in BMSCs by activating the NF-κB and STAT3 signaling pathways. We also found that BMSCs co-cultured with high IL-32- expressing MM cells produced more IL-6 compared to those co-cultured with IL-32- knockdown MM cells, indicating that MM cell secretion of IL-32 may have the same biological function as rIL-32α. It is well established that in the MM BM microenvironment, BMSCs are the primary source of IL-6, which has strong paracrine proliferation stimulation effects on the MM cells [39]. IL-6 production induced by cooperation of NF-κB and STAT3 signaling can provide feedback to keep these two signaling pathways consistently activated, thus promoting an inflammation ‘cascade amplification effect’ [40–42]. Therefore, it seems to be very important to find the key that initializes this process. Our study suggested that IL-32 secreted from MM cells may ‘start’ or at least enhance this procedure by activating NF-κB and STAT3 signaling pathways. We also confirmed that BMSCs produced-IL-6, which was induced by IL-32α feedback to promote the proliferation of MM cells. In agreement with a previous study, we have not yet found any evidence yet that IL-32α has a direct effect on MM cells (apoptosis and proliferation).

PR3, which exists in both soluble form and cell surface, is a serine protease derived from neutrophil capable of multiple biological functions. It is a major auto-antigen in Wegener's granulomatosis, a systemic vasculitic disease [43–45]. As previous studies have reported, PR3 is an IL-32 binding protein that cleaves recombinant IL-32 into separate domains and make it more active than the intrinsic protein, especially IL-32α and IL-32β [30, 38, 46]. Hence, it is considered a receptor of extracellular IL-32. In this study, our data suggested that the expression of PR3 in BMSCs was significantly increased compared with MM cells, which might be one reason that IL-32 did not show strong autocrine stimulation in MM cells but affected BMSCs. Additionally, we verified that BMSCs with low expression of PR3 reduced the effects induced by IL-32. These data indicated that the binding of IL-32 and PR3 may be critical in the production of IL-6 in the MM BM microenvironment.

Moreover, our group assayed additional cytokines induced by IL-32. In a previous study from our group, we found that MIP-1α and MIP-1β were highly expressed in myeloma cells, and MIP-1α was positively correlated with the infiltration of tumor associated macrophages [47]. IL-10 is a well-known anti-inflammatory factor involved in inflammation [48]. MIP-1α, MIP-1β and IL-10 are all primarily from MM cells or macrophages in the BM microenvironment [47–49]. Although IL-32 increased or decreased the production of these cytokines or chemokines in BMSCs, we doubt that these effects have a profound influence on MM cells, as the basement secretion in BMSCs is rather low. Interestingly, VEGF, a key cytokine mediates BM angiogenesis in MM [4], decreased significantly. Recently, Bak et al. reported that IL-32θ, a new isoform of IL-32, downregulates CCL5 expression through interaction with PKCε and STAT3 [50]. In MM, CCL5 is involved in osteolysis [51, 52]. Further studies regarding the relationships and mechanisms between VEGF, CCL5 and IL-32α in MM are underway in our group.

**Figure 8:**
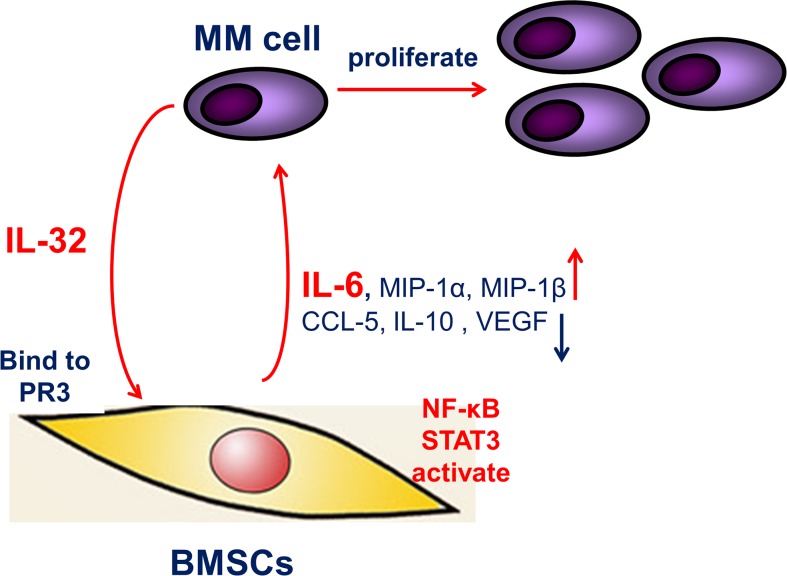
The proposed mechanism underlying the observed pro-inflammation effect of IL-32 IL-32 secreted from MM cells, increases IL-6 expression in BMSCs through activating NF-κB and STAT3 inflammation signaling pathways, thus feedback to support MM cells, resulting in the proliferation and growth of MM cells.

To summarize, our study analyzed the expression of IL-32 and its binding receptor PR3 in MM BM, and demonstrated that in the MM BM microenvironment, IL-32 induces production of IL-6 in BMSCs by activating the NF-κB and STAT3 signaling pathways, thus promoting the proliferation of MM cells (Figure 8). In expectation, IL-32 may be a new biomarker in MM diagnoses and an optimal therapy target in MM treatment.

## MATERIALS AND METHODS

### Human myeloma cell lines, primary myeloma cells, and human bone marrow stromal cells

Human MM cell lines RPMI8226, OPM2, U266, MM.1S, H929, LP-1 and ANBL-6 were kindly provided by Dr Qing Yi (Department of Cancer Biology, Lerner Research Institute, Cleveland Clinic, OH, USA) and Dr Zhiqiang Liu (Department of Pathology and Pathophysiology, Tianjin Medical University, Tianjin, China). MM cells were obtained from MM patients BM aspirates and purified by positive selection with CD138 microbeads (Miltenyi Biotech, CA, USA). A part of CD138 negative cells were collected for qRT-PCR and the rest were cultured for about 1 week. Then then suspended cells were discarded, and the adherent cells were identified by flow cytometry analysis as previously described [47, 53] and considered as BMSCs.

BM aspirates, BM biopsies and blood samples were obtained from newly diagnosed untreated MM patients and healthy donors. All human participants provided written informed consent, and the study protocol was approved by the Ethics Committee of the First Affiliated Hospital, Zhejiang University School of Medicine. The plasma of BM aspirates and serum were used for ELISA detection of cytokines. Cells from BM biopsies were used for immunofluorescence analysis.

### Cell culture

RPMI8226, OPM2, H929, LP-1, ANBL-6, MM BMSCs and primary MM cells were cultured in RPMI 1640 medium (Thermo Scientific, HyClone) containing 10% fetal bovine serum (FBS) (Thermo Fisher Scientific, Gibco), and 1% L-glutamine at 37°C and 5% CO_2_.

Bone marrow stromal cells conditioned medium (BCCM) was prepared as follows: bone marrow stromal cells were cultured at a density of 2×10^5^/mL in RPMI-1640 containing 10% FBS in the presence of 0-80 ng/mL rIL-32α. Culture medium was collected after 24 h and stored at 4°C for up to 1 wk before use. We diluted BCCM with fresh complete medium (50% BCCM to 50% complete medium) to apply for further studies.

When BMSCs reached 70% confluence in 24-well plates, MM cell lines (2×10^5^ cells per well) were put in Transwell inserts (pore size: 0.4μm, Corning) and co-cultured with BMSCs for 24, 48 and 72h. Then, culture medium supernatant was prepared for ELISA, or 100 μL MM cells suspension was aspirated for CCK8 analysis.

### Reagents and antibodies

Recombinant human IL-32α (rIL-32α), Recombinant human IL-6 (rIL-6) and neutralization antibodies to human IL-6 were purchased from R&D systems (Minneapolis, MN, USA). Antibodies to human IL-32, PR3, and immunofluorescence antibody to IL-32 were purchased from Abcam (Cambridge, UK). Western blot antibodies to STAT3, pSTAT3 Ser-727, pSTAT3 Tyr-727, p38MAPK, pp38MAPK, p65 and pp65 were purchased from Cell Signaling Technology (Danvers, MA, USA). NF-κB inhibitor QNZ and STAT3 inhibitor BP-1-102 were purchased from Selleckchem (Houston, TX, USA). Primary antibody against GAPDH was purchased from Sigma-Aldrich. Horseradish peroxidase (HRP)-conjugated anti-rabbit and anti-mouse antibodies were purchased from Jackson Immuno research Laboratories (West Grove, PA, USA).

### Enzyme-linked immunosorbent assay (ELISA) and cytokine array

A Human IL-32 Duoset ELISA kit was purchased from R&D systems (Minneapolis, MN, USA). IL-6 ELISA and TNF-α ELISA kits were purchased from DAKEWE (Beijing, China). Cytokines were detected by following the manufacturer's instructions. Cytokine array technology and analysis was provided by RayBiotech (Guangzhou, China).

### Cell proliferation assays

A CCK8 assay was used to detect MM cells and BMSCs proliferation. MM cells (1-2×10^4^ cells per well) or BMSCs (2-4×10^3^ cells per well) were cultured in 96-well plates for 24, 48 and 72 h, with or without stimulation of rIL-32α; preparation of MM cells co-cultured with BMSCs was mentioned above. Cells were treated with 10μL of CCK8 solution and incubated at 37°C for another 1-2h. A microplate reader (Bio-Rad, Model 680) was used to measure the absorbance at 450 nm. Cell viability (%) = OD value of test sample/ OD value of control ×100%.

### Immunofluorescence

Cells from BM biopsies were seeded on cover glass and fixed in 4% paraformaldehyde for 30 min in 4°C. Then, 0.3% Triton X-100 was used to permeabilize cells for 10 min. After three washes with PBS, the cells were immunostained with anti-IL-32 and anti-CD138 (Abcam, Cambridge, UK) at 4°C overnight. Subsequently, after washing, cells were incubated with goat anti-mouse IgG (Invitrogen, USA) in the dark. Then, cells were washed again and incubated in the dark with DAPI (Wako, Osaka, Japan) for 5min. Finally, photos were taken using fluorescence microscopy (Olympus, Tokyo, Japan).

### RT-PCR

Total RNA was isolated using RNAiso^TM^ Plus (TaKaRa, Shiga, Japan) according to the instructions. cDNA was generated using a PrimeScript^TM^ T Reagent Kit with DNA Eraser (TaKaRa, Shiga, Japan). RT-PCR was performed in a GeneAmp PCR System 9700 (Applied Biosystems, Thermo Fisher Scientific, MA, USA), and quantitative RT-PCR was performed in a CFX96 Real-Time System (Bio-Rad, CA, USA), following the manufacturer's instructions. The primer sequences for IL-32(total) were (forward) 5′-AAGCTGAAGGCCCGAATG-3′ and (reverse) 5′-CCTCGGCACCGTAATCCA-3′. The primer sequences for IL-32α were (forward) 5′-GCTGGAGGACGACTTCAAAGA-3′ and (reverse) 5′- GGGCTCCGTAGGACTTGTCA-3′. The primer sequences for IL-32β were (forward) 5′-CAGTGGAG CTGGGTCATCTCA-3′ and (reverse) 5′- GGGCCT TCAGCTTCTTCATGTCATCA-3′. The primer sequences for IL-32γ were (forward) 5′-AGGCCCGAATGGTAAT GCT-3′ and (reverse) 5′- CCACAGTGTCCTCAGTGT CACA-3′. The primer sequences for PR3 were (forward) 5′-CACTGCCTGCGGGACATA-3′ and (reverse) 5′-ACACCTGAGCCACCGAGAA-3′.

### Western blot analysis

Cells were collected and extracted with RIPA buffer. Supernatants were collected for Western blot. Briefly, 20-40 μg of proteins were separated by 8%-12% sodium dodecyl sulfate-polyacrylamide gel electrophoresis and transferred to polyvinylidene difluoride membranes (Merck Millipore, Germany). The membranes were blocked with 5% bovine serum albumin for 1-2 h and then incubated with primary antibodies overnight at 4°C. The membranes were washed the next day with Tris-buffered saline with Tween 20 (TBS-T) and then incubated with HRP-conjugated anti-rabbit or anti-mouse antibodies for 2 h at room temperature. The membranes were washed with TBS-T again, and protein bands were detected using a ChemiDoc^TM^ MP Imaging System (Bio-Rad) with an enhanced chemiluminescence detection kit for HRP (Biological Industries, Israel, Beit Haemek Ltd.).

### Transfection of BMSCs and MM cells

BMSCs were cultured in 12-well plates (2×10^5^ cells per well) the day before transfection. When cells reached 70% confluence, they were transfected with PR3 siRNA (Vigene Biosciences, Rockville, USA) (forward, 5′-CGGAGAACAAACUGAACGA-3′, and reverse, 5′-UCGUUCAGUUUGUUCUCCG-3′) using lipofectamine^TM^ 2000 (Invitrogen, USA). Control group of MM cells were transfected with siRNA (Vigene Biosciences, Rockville, USA) (forward, 5′-UUCUCCGAACGUGUCACGU-3′, and reverse, 5′-ACGUGACACGUUCGGAGAA-3′). After 6 h, the transfection medium (Opti-MEM) was removed and replaced with fresh complete medium. After 48-72 h, BMSCs were collected and Western blot was used to verify the efficiency of the transfection.

MM cells were seeded in 24-well plates (5×10^4^ cells per well) and transfected with IL-32 shRNA (Vigene Biosciences, Rockville, USA) (GATCCGTGACAAGGTCATGAGATGGTTCTTCAAGAGAGAACCATCTCATGACCTTGTCACTTTTTTA) using pLent-U6-GFP-Puro vectors. Control group of MM cells were transfected with empty vectors. After 5-7 days, MM cells were collected and cultured in medium with puromycin (1 μg/mL) to select for well-transfected MM cells. After another 1-2 weeks, Western blot was used to verify the efficiency of transfection.

### Statistical analysis

Results are presented as the mean±SD of at least three independent experiments. Two-tailed Student's t-test was used to analyze significant differences between two groups and one-way ANOVA was used to estimate between three or more groups. *p* values <0.05 were considered statistically significant. All analyses were performed using GraphPad Prism 5.0 (GraphPad Software, CA, USA).

## SUPPLEMENTARY MATERIALS FIGURE


