# Supplementary material for: Interleukin-32α promotes the proliferation of multiple myeloma cells by inducing production of IL-6 in bone marrow stromal cells
Source: Oncotarget. 2017 Oct 7;8(54):92841–54. doi: 10.18632/oncotarget.21611 (PMC5696226; doi:10.18632/oncotarget.21611)
Supplement: Supplementary file 1 [file oncotarget-08-92841-s001.pdf]

# Interleukin-32α promotes the proliferation of multiple myeloma cells by inducing production of IL-6 in bone marrow stromal cells

## SUPPLEMENTARY MATERIALS

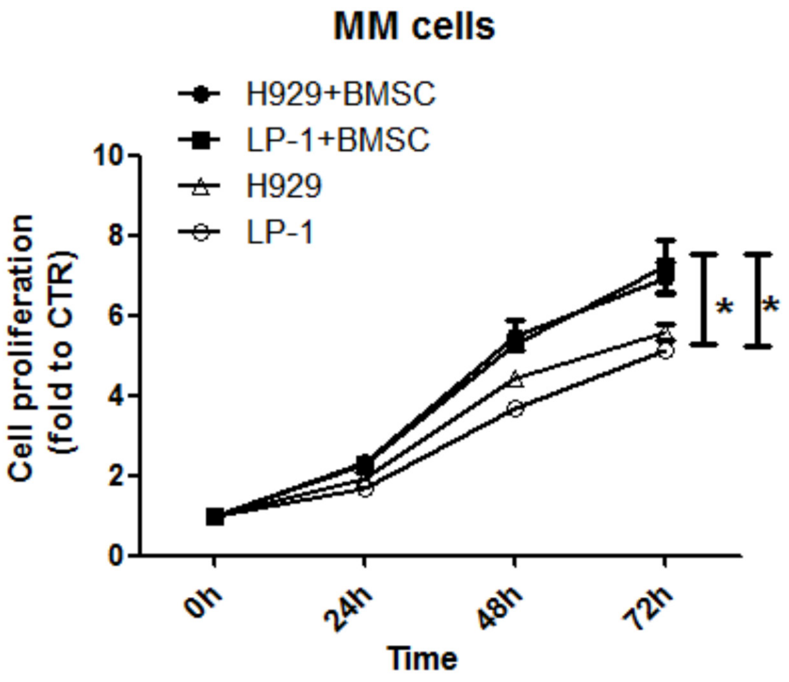


**Supplementary Figure 1: Cell proliferation in IL-32 low-expression MM cell lines, H929 and LP-1, cultured alone or co-cultured with BMSCs for 24, 48, and 72 h.** MM cells were co-cultured with BMSCs in 24-well plates and transferred to 96-well plates to be assayed. Repeated in three independent experiments, measured by CCK8.
